# Effect of the standard herbal preparation, STW5, treatment on dysbiosis induced by dextran sodium sulfate in experimental colitis

**DOI:** 10.1186/s12906-021-03337-8

**Published:** 2021-06-08

**Authors:** Sarah S. Mohamed, Nourtan F. Abdeltawab, Walaa Wadie, Lamiaa A. Ahmed, Ramy M. Ammar, Sabine Rabini, Heba Abdel-Aziz, Mohamed T. Khayyal

**Affiliations:** 1 Department of Pharmacology and Toxicology, Faculty of Pharmacy, Cairo University, Kasr El-Aini Street, Cairo, 11562 Egypt; 2 Department of Microbiology and Immunology, Faculty of Pharmacy, Cairo University, Cairo, Egypt; 3 Bayer Consumer Health, Steigerwald Arzneimittelwerk GmbH, Darmstadt, Germany; 4 Pharmacology Department, Faculty of Pharmacy, Kafrelsheikh University, Kafrelsheikh, Egypt

**Keywords:** Dysbiosis, Gut microbiota, STW 5, Ulcerative colitis

## Abstract

**Background:**

The standardized herbal preparation, STW 5, is effective clinically in functional gastrointestinal disorders and experimentally in ulcerative colitis (UC). The present study explores whether the beneficial effect of STW 5 involves influencing the intestinal microbiota.

**Methods:**

UC was induced in Wistar rats by feeding them 5% dextran sodium sulfate (DSS) in drinking water for 7 days. Rats were treated concurrently with STW 5 and sacrificed 24 h after last drug administration. Fecal samples were used to determine changes in the abundance of selected microbial phyla and genera using real-time PCR.

**Results:**

Induction of UC led to dysbiosis and changes in the gut microbiota. The changes included an increase in some genera of the Firmicutes, namely Enterococcus, and a decrease in others, namely Blautia, Clostridium, and Lactobacillus. DSS further induced a marked increase in the abundance of Bacteroidetes and Proteobacteria as well as in the relative abundance of Actinobacteria and its genus Bifidobacterium. Methanobrevibacter levels (phylum Euryarchaeota) were also increased. Microbial dysbiosis was associated with changes in various parameters of colonic inflammation. STW 5 effectively guarded against those changes and significantly affected the indices of edema and inflammation in the UC model. Changes in colon length, colon mass index, inflammatory and apoptotic markers, and histological changes induced by DSS were also prevented.

**Conclusions:**

Dysbiosis plays a contributing role in the development of DSS-induced UC. Derangements in the microbial flora and associated inflammatory processes were largely prevented by STW 5, suggesting that this effect might contribute towards its beneficial usefulness in this condition.

**Supplementary Information:**

The online version contains supplementary material available at 10.1186/s12906-021-03337-8.

## Background

Gastrointestinal diseases (GID), whether inflammatory or functional (FGID), affect people worldwide and impair their quality of life and work productivity [1]. Inflammatory bowel diseases (IBD) are usually manifested either as ulcerative colitis (UC) or as Crohn’s disease (CD) [2]. Colitis induced by dextran sodium sulfate (DSS) in rats mimics the clinical and histological features of UC by interfering with intestinal barrier function and stimulating local inflammatory processes [3]. Growing evidence further suggests the involvement of gut microbiota in IBD [4]. It has been postulated that the gut microbiota imbalance (dysbiosis) could initiate immune responses by compromising the mucosal barrier and stimulating local and systemic immunity [5, 6]. This fact qualifies the DSS model to be used as a dysbiosis model [7].

Furthermore, altered motility, visceral hypersensitivity, immune alterations, low-grade inflammation, dysfunctional brain-gut axis, and compromised epithelial barrier function have all been postulated to contribute to the symptoms in functional dyspepsia (FD) and irritable bowel syndrome (IBS) [8, 9]. Gut microbiota has been shown to modulate many of these physiological functions [10, 11]. Although no consistent microbial signature has been associated with FGIDs, several lines of evidence support a role for gut microbes in the development of FGID symptoms [11].

Gut microbiota is a complex ecosystem dominated by four main phyla: *Proteobacteria, Bacteroidetes, Firmicutes, and Actinobacteria* [12, 13]. In a healthy state, the gut microbiota has a mutualistic relationship with the human host. The host intestine provides the microbes with a niche and the microbial ecosystem contributes to maintaining homeostasis by modulating several physiological functions such as nutrient digestion, immune responses, and normal perception of visceral pain [14].

STW 5 (Iberogast®) is a standardized multi-component herbal preparation consisting of a combination of nine medicinal herbal extracts, commercially available in Europe. It was shown to be effective in FD and IBS in several randomized clinical studies [15] and was previously reported to have anti-ulcerogenic and mucosal protective effects as well as potent anti-inflammatory properties [16]. The present study aimed at exploring whether the beneficial effect of STW 5 could also involve modulation of the intestinal microbiota.

## Methods

### Animals

Adult female Wistar rats, weighing 150–200 g each, were obtained from the Modern Veterinary Office for Laboratory Animals, Cairo, Egypt. Rats were provided with a standard pellet diet and were given water ad libitum. The animals were housed at a temperature of 22 ± 3 °C and a 12-h light/dark cycle as well as at a constant relative humidity throughout the experimental period. Animals were left to acclimatize for at least 7 days before subjecting them to experimentation.

The study was carried out in compliance with the ARRIVE guidelines and experimental procedures were approved by the institutional Ethical Committee for Animal Experimentation at the Faculty of Pharmacy, Cairo University, Cairo, Egypt, approval number (PT 1769) following the guidelines laid out in the Guide for the Care and Use of Laboratory Animals, National Academy of Science.

### Drugs

STW5 (Iberogast®) is a commercially available standardized herbal preparation that was generously provided by Bayer consumer health (Darmstadt, Germany). It consists of hydroethanolic extracts of *Iberis amara L. (Brassicaceae)* (15%), *Melissa officinalis L. (Lamiaceae)* (10%), *Matricaria chamomilla (Compositae)* (20%), *Carum carvi L. (Apiaceae)* (10%), *Mentha piperita L. (Lamiaceae)* (5%), *Angelica archangelica L. (Apiaceae)* (10%), *Silybum marianum (L.) Gaertn. (Compositae)* (10%), *Chelidonium majus L. (Papaveraceae)* (10%), and *Glycyrrhiza glabra L. (Leguminosae)* (10%). The preparation and every single extract were well characterized according to the guidelines of the European Medicines Agency. The extraction processes as well as the quality controls were previously described in detail [17]. Briefly, the extracts were prepared, and quality controlled according to Good Manufacturing Practice and Good Agricultural Practice of Medicinal and Aromatic Plants. The quality of each extract was tested according to individual specifications as chromatographic fingerprint [17, 18].

### Induction of colitis

Colitis was induced in rats by adding DSS, molecular weight 37–40 kD, (TdB Consultancy, Uppsala, Sweden), to the drinking water in a concentration of 5% (w/v) for 1 week [19].

### Experimental design

Adult rats were randomly allocated to three groups of 14–16 animals each as follows:

- Vehicle control group: received normal tap water (without DSS) and given 31% ethanol (STW 5 vehicle) 5 mL/kg, orally daily for 1 week.
- UC group: received 5% DSS in drinking water and given concurrently 31% ethanol (STW 5 vehicle) 5 mL/kg, orally daily for 1 week.
- UC/STW 5 group: received 5% DSS in drinking water and given concomitantly STW 5 (5 mL/kg), orally daily for 1 week. This dose was chosen after carrying out preliminary experiments with two doses (2 ml and 5 ml /kg) of the preparation and selected on the basis that it had more consistent effects on the intestinal microbiota.

Twenty-four hours after the last drug administration, rats were euthanized using halothane anesthesia followed by cervical dislocation and the colon and caecum from all animals were excised. The colon length was measured, rinsed in ice-cold saline, cleaned of extraneous tissue, dried on filter paper, and weighed. The ratio of colon weight in milligrams to the total body weight in grams was taken as the colon mass index and was used as a measure of the degree of colonic edema and severity of inflammation. The colon was then cut longitudinally into two segments: one was fixed in 10% formalin for histological examination, and the other was homogenized in ice-cold saline to obtain a 10% homogenate for the assessment of biochemical parameters. The entire caeca were stored at -20 °C until further use. Fecal samples from each caecum were used for microbial genomic DNA isolation and further analysis.

### Determination of biochemical parameters associated with colitis

The colon homogenate was centrifuged at 6000 rpm for 30 min at 4 °C. The supernatant was used for assaying tumor necrosis factor-alpha (TNF-α), nuclear factor kappa B (NFκB), and caspase-3 using rat specific enzyme-linked immunosorbent assay (ELISA) kits from Elabscience Biotechnology Co. (Texas, USA), Hangzhou Eastbiopharm Co. (Hangzhou, China) and Cloud-Clone corp. (Texas, USA), respectively.

### Microbial genomic DNA isolation

The excised frozen caeca were subjected to fecal genomic DNA extraction using Zymo research fecal DNA extraction kit (Zymo Research Corp., Irvine, CA, USA) following the manufacturer’s protocol. This protocol was effective in removing traces of DSS from the isolated DNA, as DSS is a PCR inhibitor [20]. DNA concentration was determined by measuring the absorbance at 260 nm using Implen nanophotometer P-330 (Implen GmbH, Munich, Germany).

### Relative abundance of microbial phyla using quantitative real-time PCR

The changes in the main gut-associated microbiota were quantified using specific primers targeting different microbial genera 16S ribosomal ribonucleic acid (*rRNA*) gene by Real Time-PCR (qPCR) as described in Table 1. qPCR experiments were performed using QuantiFast SYBR Green PCR Kit (Qiagen, Hilden, Germany) on a Rotor-Gene Real-Time PCR machine (Qiagen, Hilden, Germany). The thermal cycling conditions were optimized as follows: an initial DNA denaturation step at 95 °C for 10 min, followed by 40 cycles of denaturation at 95 °C for 10 s, primer annealing at optimal temperature for 20 s, extension at 72 °C for 15 s. Melt curve analysis was performed by slowly cooling from 95 °C to 60 °C (0.05 °C per cycle) with simultaneous measurement of the SYBR Green I signal intensity. All PCR tests were carried out in duplicates of each group pool.

**Table 1:** Primer sequences and annealing temperatures for selected analyzed microbial phyla and genera *16 s rRNA* gene

Microbial phyla and genera	*16 s rRNA* Gene Primer Sequence 5′ – 3′	Annealing Temperature (°C)	Reference
***Firmicutes*** **phylum**	ATG TGG TTT AAT TCG AAG CA	60	[21]
	AGC TGA CGA CAA CCA TGC AC		
*Lactobacillus* genus	GAG GCA GCA GTA GGG AAT CTT C	53	[22]
	GGC CAG TTA CTA CCT CTA TCC TTC TTC		
*Clostridium* genus	GCA CAA GCA GTG GAG T	50	[23]
	CTT CCT CCG TTT TGT CAA		
*Blautia* genus	CGG TAC CTG ACT AAG AAG C	55	[24]
	AGT TTC ATT CTT GCG AAC G		
*Enterococcus* genus	CCC TTA TTG TTA GTT GCC ATC ATT	61	[24]
	ACT CGT TCT TCC CAT GT		
***Bacteroidetes*** **phylum**	CAT GTG GTT TAA TTC GAT GAT	60	[21]
	AGC TGA CGA CAA CCA TGC AG		
*Bacteroides* genus	GAG AGG AAG GTC CCC CAC	60	[21]
	CGC TAC TTG GCT GGT TCA G		
*Prevotella* genus	GGT TCT GAG AGG AAG GTC CCC	55	[25]
	TCC TGC ACG CTA CTT GGC TG		
***Actinobacteria*** **phylum**	CGC GGC CTA TCA GCT TGT TG	57	[26]
	CCG TAC TCC CCA GGC GGG G		
*Bifidobacterium* genus	CTC CTG GAA ACG GGT GG	55	[23]
	GGT GTT CTT CCC GAT ATC TAC A		
***Proteobacteria*** **phylum**	CAT GAC GTT ACC CGC AGA AGA AG	63	[27]
	CTC TAC GAG ACT CAA GCT TGC		
***Fusobacteria*** **phylum**	CCC TTC AGT GCC GCA GT	51	[27]
	GTC GCA GGA TGT CAA GAC		
***Euryarchaeota*** **phylum** *Methanobrevibacter* genus	CCG GGT ATC TAA TCC GGT TC	50	[28]
	CTC CCA GGG TAG AGG TGA AA		

### Microbial genomic DNA standard curves

Microbial genomic DNAs used for the construction of the standard curves were extracted from *Lactobacillus acidophilus* (ATCC 4356) and *Enterococcus faecalis* (ATCC 19433) obtained from the American type culture collection (Virginia, USA). The genomic DNA of *Escherichia coli* (DSM 498), *Prevotella intermedia* (DSM 20706), *Blautia producta* (DSM 2950), *Bacteroides vulgatus* (DSM 1447), *Methanobrevibacter smithii* (DSM 861), *Ilyobacter polytropus* (DSM 2926), *Clostridium leptum* (DSM 753) and *Bifidobacterium bifidum* (DSM 20456) were obtained from Leibniz Institute DSMZ-German Collection of Microorganisms and Cell Cultures, Braunschweig, Germany. Standard curves were constructed for each experiment using serial ten-fold dilution of microbial genomic DNA of the mentioned standard cultures of microbes, corresponding to 30 to 3 × 10^6^ *16S rRNA* gene copies. The mass for one microbial genome was calculated by using Avogadro’s number and assuming the mean molecular weight of a base pair to be 660 g/mol. Standard curves were normalized to the copy number of the *16S rRNA* gene for each microbial species. The microbial concentration from each fecal sample was calculated by comparing the cycle threshold (Ct) values obtained from the standard curves and expressed as gene copies/ gram of feces.

### Histopathological examination of the colon

Transverse sections, 4–6 μm thin, were prepared from paraffin-embedded colon segments from each animal of all experimental groups. The sections were stained with hematoxylin and eosin (H&E) and examined under 200 magnification using a light microscope by a pathologist blinded to the treatment regimens.

### Statistical analysis

All data obtained were presented as means ± SEM. Results were analyzed using a one-way analysis of variance test (one-way ANOVA) followed by Tukey’s multiple comparison test. Statistical analysis was performed using Graph Pad Prism software (version 6.04). For all the statistical tests, the level of significance was taken at *p* < 0.05.

## Results

### Effect on colon length, colon mass index, colon histology, inflammatory and apoptotic biomarkers

**Fig. 1:**
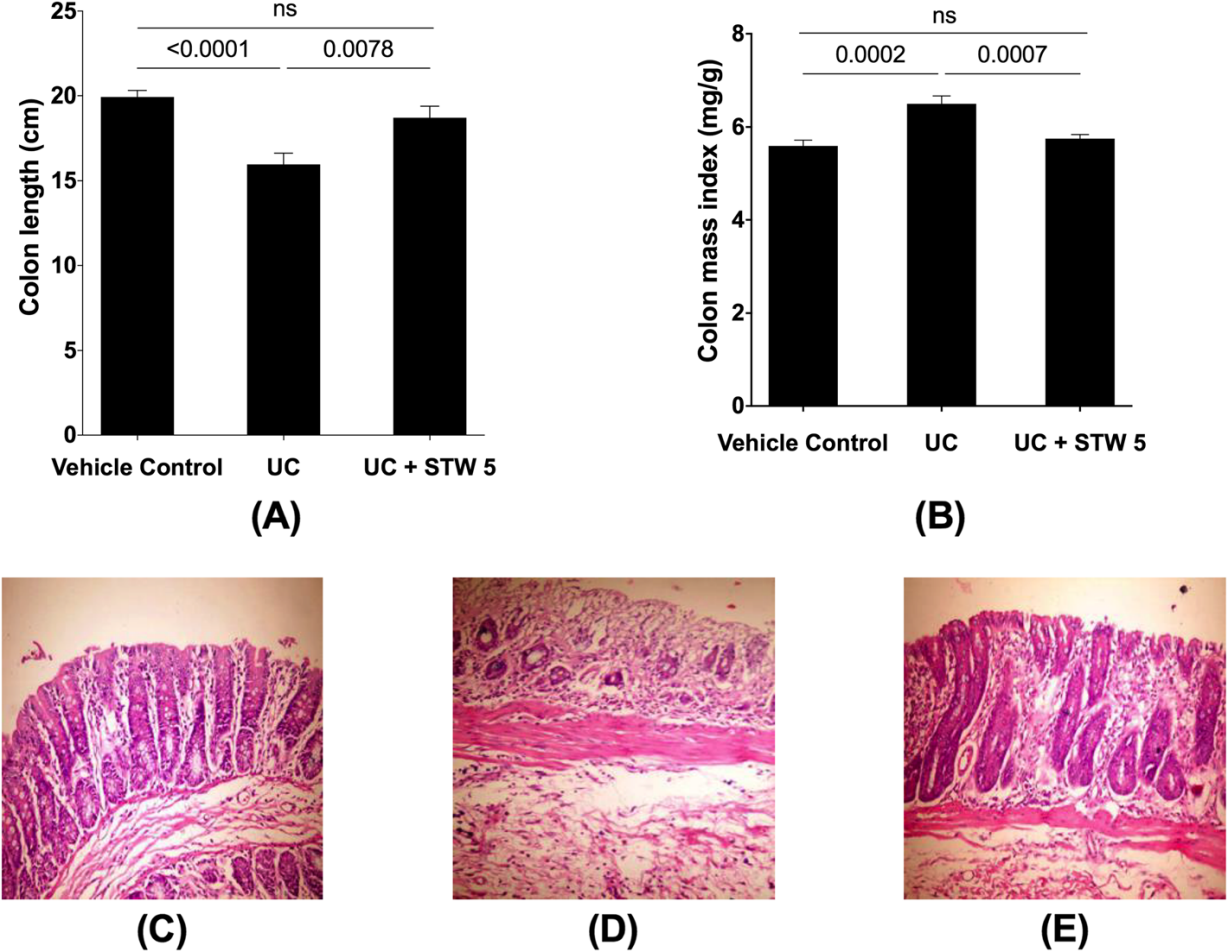
Effect of STW 5 treatment on colon length, colon mass index, and histopathological changes, in colonic tissue of rats with DSS-induced colitis. Induction of colitis led to a reduction in colon length **A** as compared to the vehicle control group and this was associated with an increase in colon mass index **B**. Normal histological structure of colonic mucosa in normal control rats **C**. Colon of rats with DSS-induced colitis showing necrosis of epithelium, distortion of crypts, inflammatory infiltrate in lamina propria as well as sub-mucosal edema **D**. Apparently normal mucosa in the colon of rats with DSS-induced colitis after treatmet with STW 5 which tended to prevent these changes **E**. Data represented as means ± standard deviation of at least two independent experiments with number of animals of at least 14 animals per group

**Fig. 2:**
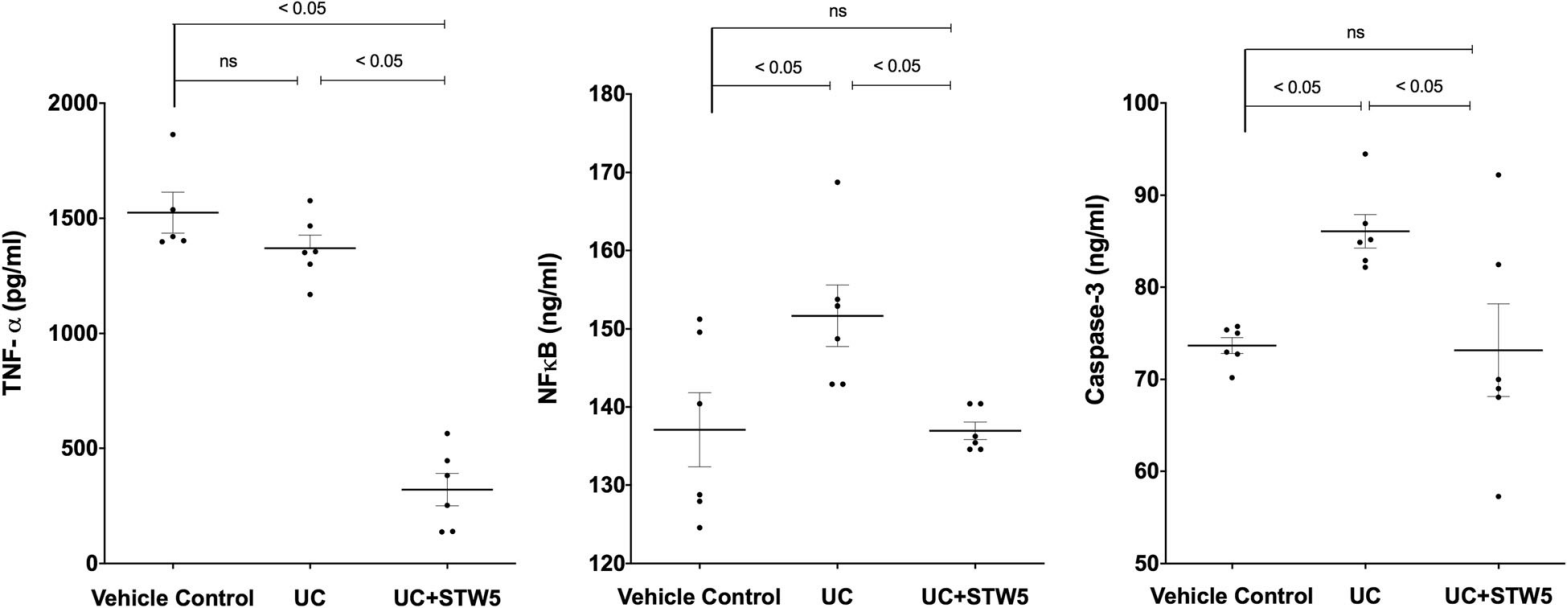
Effect of STW 5 treatment on inflammatory and apoptotic biomarkers in colonic tissue of rats with DSS induced UC. TNF-α, **A**, NFκB **B**, and caspase-3 **C** measurd by ELISA were significantly elevated in DSS-induced colitis, but this rise was prevented by STW 5 treatment. Data represented as means ± standard deviation of at least two independent experiments with number of animals of 6 animals per group

Induction of colitis led to a reduction in rat colon length by approximately 20% (Fig. 1a). This was associated with an 18% increase in colon mass index (Fig. 1b). Histological photomicrographs of control colons showed well-defined crypt lengths and no edema in the mucosa and submucosa (Fig. 1c). However, DSS-treated animals (Fig. 1d) showed loss of epithelial cell and crypt architecture, inflammatory cell infiltration with marked necrosis of the epithelium, and submucosal edema. Treatment with STW 5 (Fig. 1e) largely protected against these histological changes. Furthermore, DSS induced changes in various parameters indicative of inflammation and apoptosis as evidenced by a marked elevation in the colonic content of TNF-α, NFκB (Fig. 2 a and b), and caspase-3 level (Fig. 2 c). These changes tended to be prevented by treatment with STW 5.

### Effect on intestinal microbiota

**Fig. 3:**
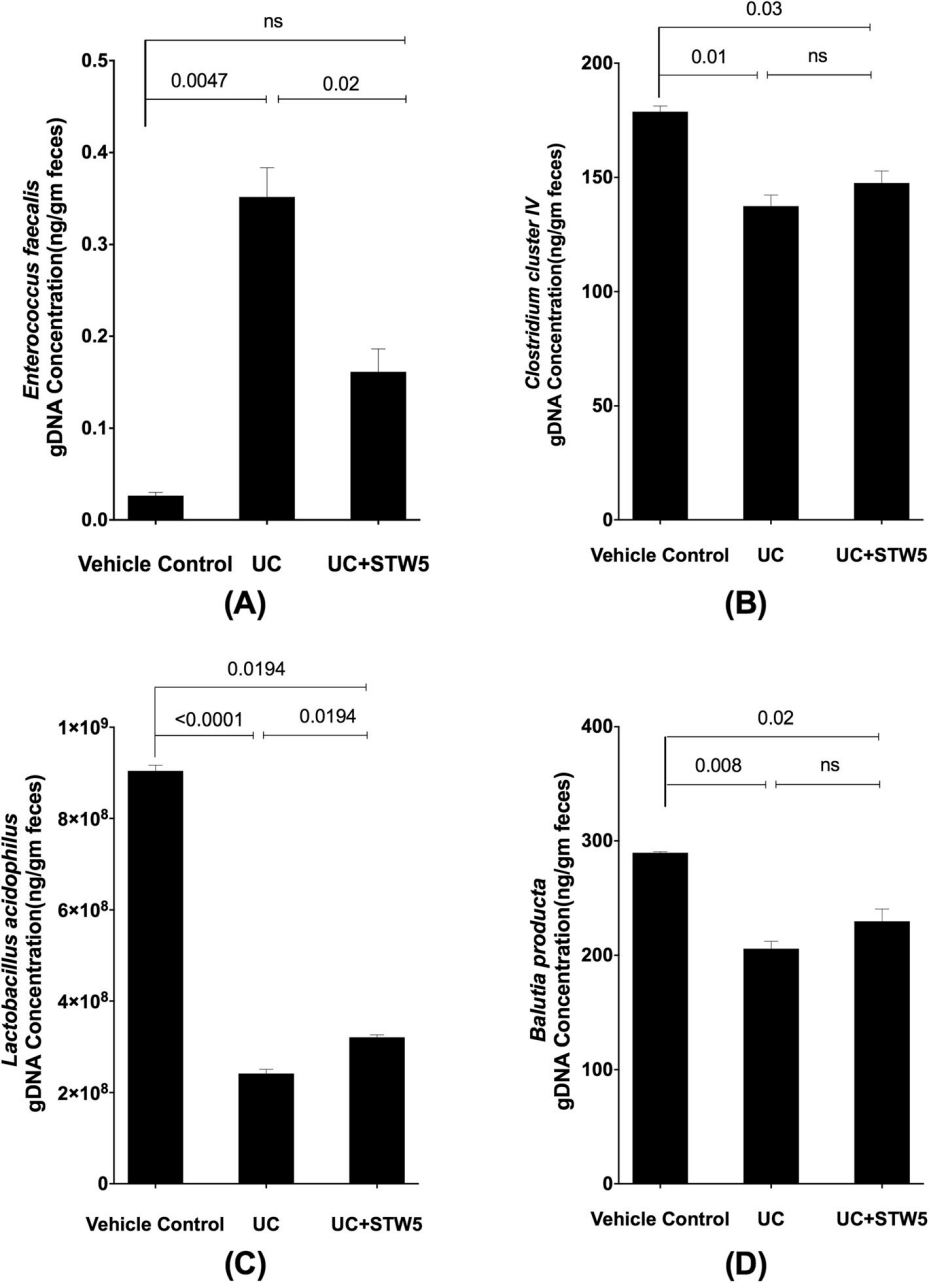
Effect of STW 5 treatment on the relative abundace of *Firmicutes* phylum, *Enterococcus* **A**, *Clostridium* **B**, *Lactobacillus* **C**, *Balutia* **D** in rats with DSS induced UC. Members of *Firmicutes* phylum were affected differently. *Enterococcus* population showed 15-fold increase as compared to vehicle control group and STW 5 treatment completely normalized its level. On the other hand, *Clostridium, Lactobacillus and Blautia* decreased by 1.3, 3.7 and 1.4 folds (23, 73 and 28%), respectively. STW 5 tended to slightly increase these levels. The microbial concentration expressed as ng per gram of feces was calculated taking *16S rRNA* gene copy number into consideration as detailed in the Methods section. Data represented as means ± standard deviation of at least two independent experiments with number of animals of at least 14 animals per group

**Fig. 4:**
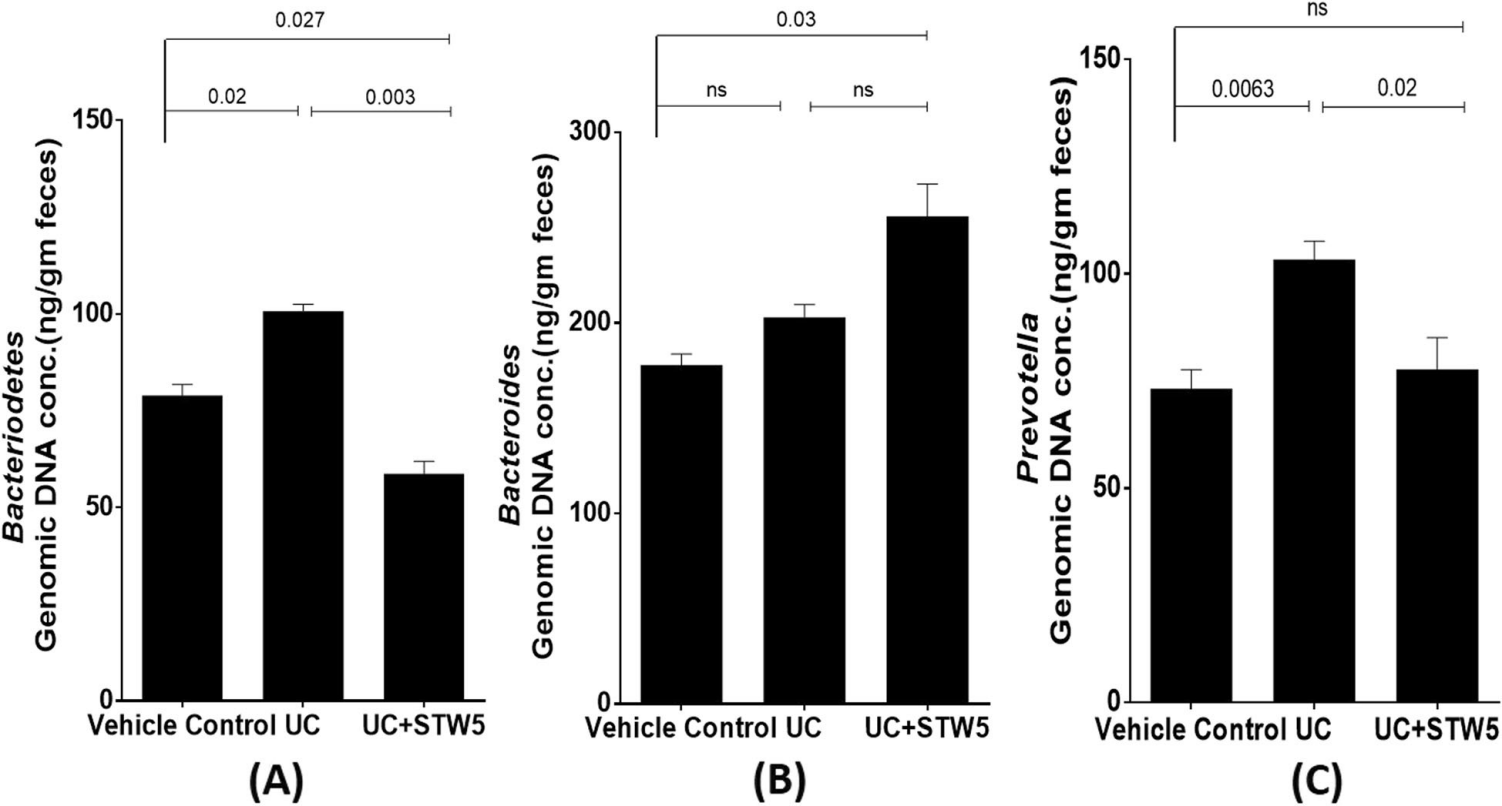
Effect of STW 5 treatment on the relative abundance of *Bacteriodetes* phylum **A**, *Bacteroides* **B**, *Prevotella* **C** in rats with DSS induced UC. All members of *Bacteriodetes* phylum showed a significant increase in DSS-induced colitis model. STW 5 succeeded to significantly reverse these changes. The microbial concentration expressed as ng per gram of feces was calculated taking *16S rRNA* gene copy number into consideration as detailed in methods section. Data represented as means ± standard deviation of at least two independent experiments with number of animals of at least 14 animals per group

**Fig. 5:**
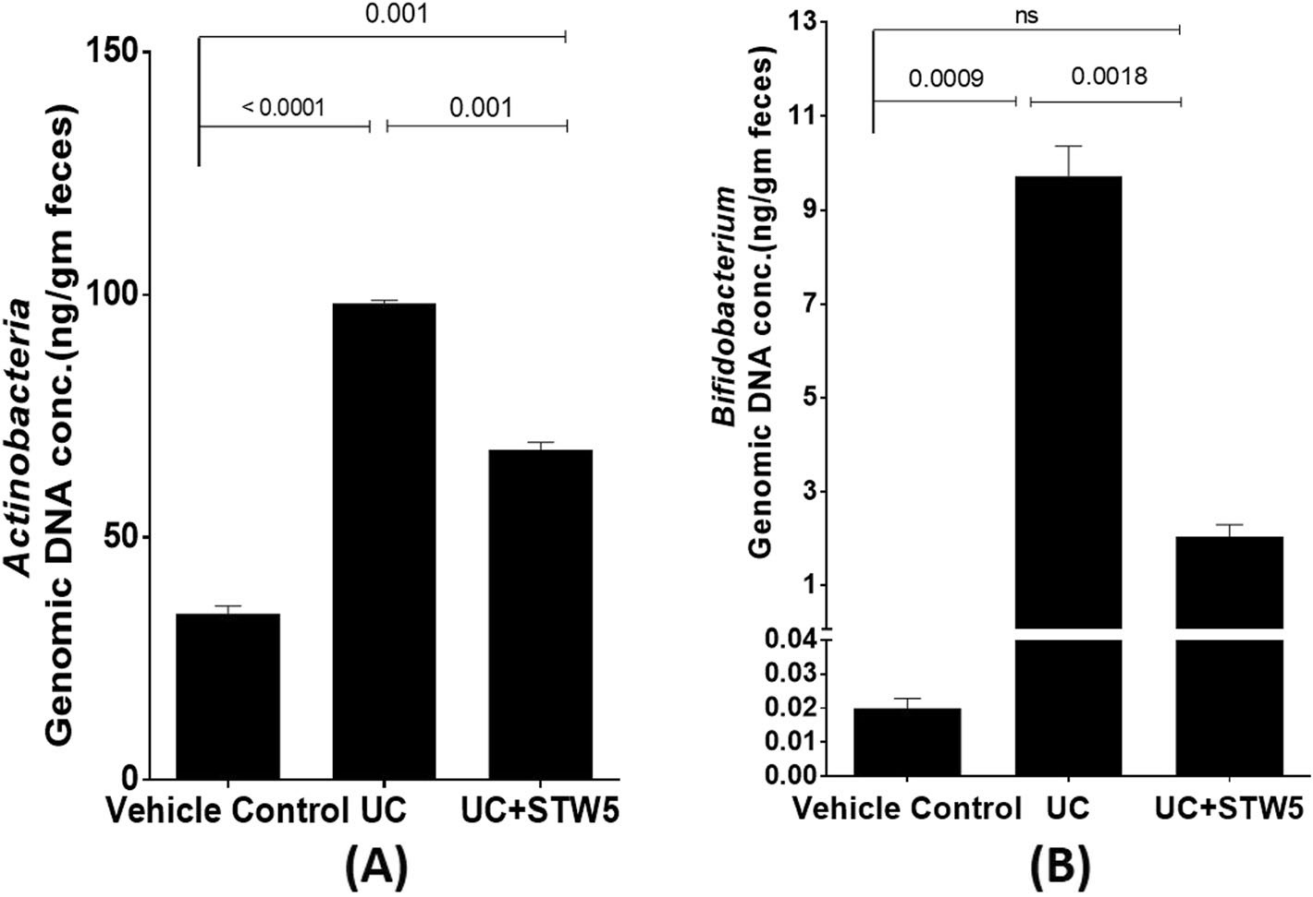
Effect of STW 5 treatment on the relative abundance of *Actinobacteria* **A** and *Bifidobacterium* **B** in rats with DSS induced UC. *Actinobacteria* and *Bifidobacterium* displayed the same pattern of change. DSS induced colitis led to a 2.8 and 486-fold increase in *Actinobacteria* and *Bifidobacterium* respectively, an effect which was significantly reversed by STW 5 treatment. The microbial concentration expressed as ng per gram of feces was calculated taking *16S rRNA* gene copy number into consideration as detailed in the Methods section. Data represented as means ± standard deviation of at least two independent experiments with number of animals of at least 14 animals per group

**Fig. 6:**
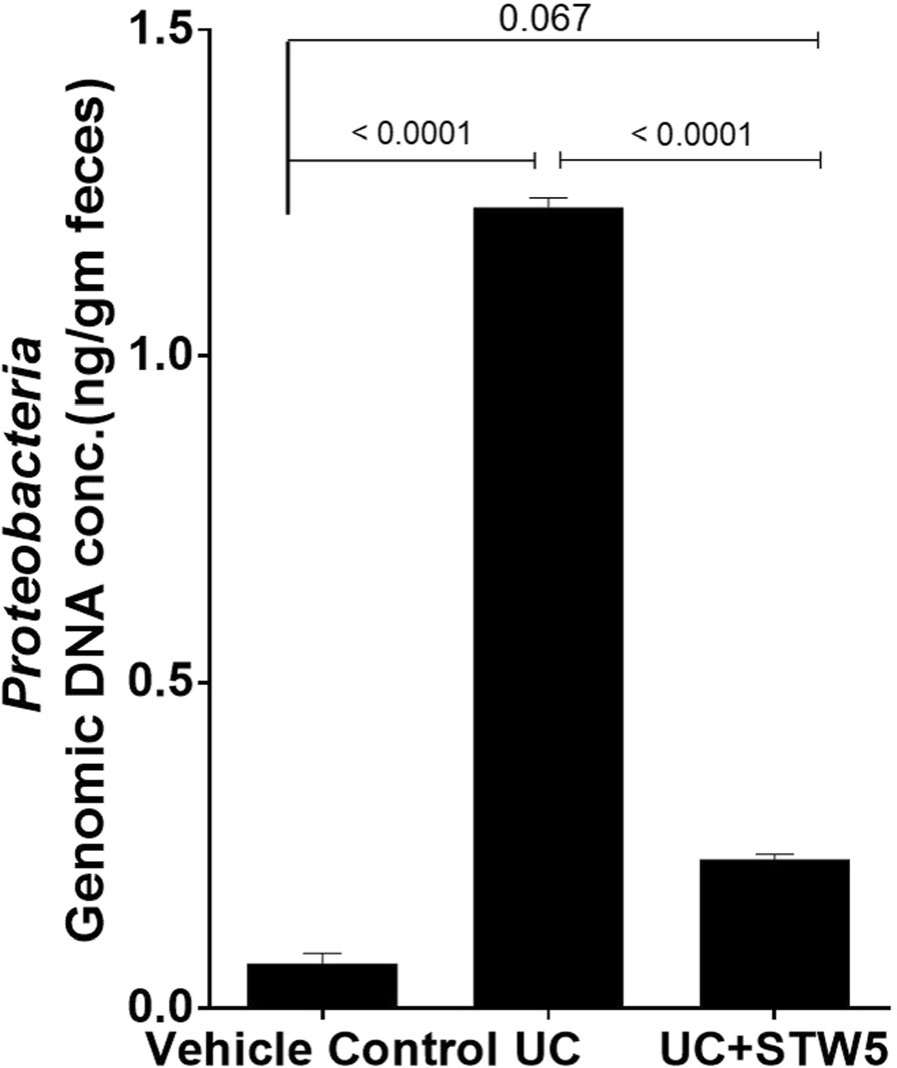
Effect of STW 5 treatment on the relative abundance of *Proteobacteria* in rats with DSS induced UC. DSS administration led to a 20-fold increase in *Proteobacteria* relative abundance which was significantly reduced after STW 5 treatment. The microbial concentration expressed as ng per gram of feces was calculated taking *16S rRNA* gene copy number into consideration as detailed in the Methods section. Data represented as means ± standard deviation of at least two independent experiments with number of animals of at least 14 animals per group

**Fig. 7:**
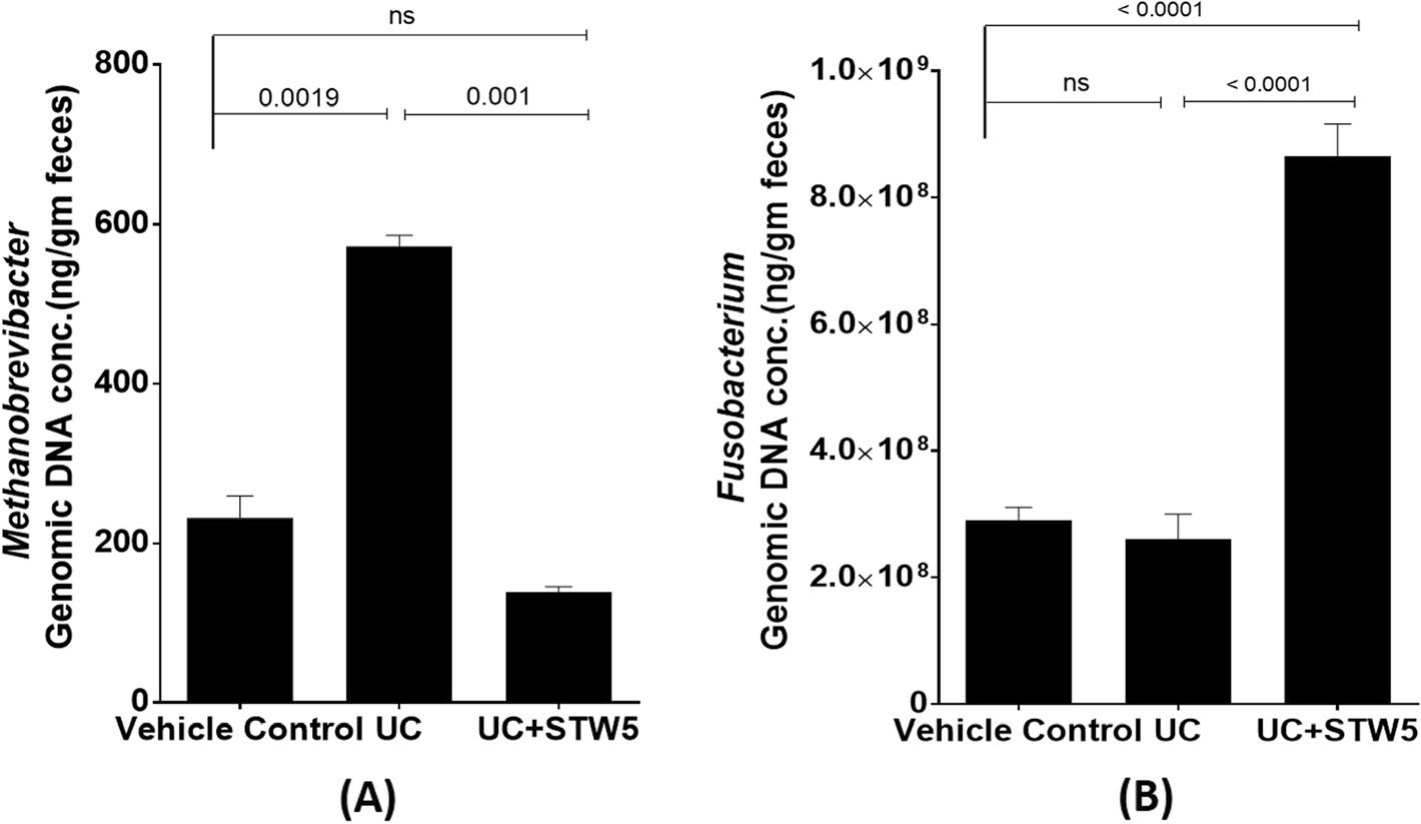
Effect of STW 5 treatment on relative abundance of *Fusobacterium* and *Methanobrevibacter* in rats with DSS induced UC. DSS administration increased *Methanobrevibacter* **A** by approximately 2.5 folds compared to vehicle control group. STW 5 succeeded to restore *Methanobrevibacter* to its normal levels. Meanwhile, DSS slightly decreased *Fusobacterium* **B** population and increased levels were caused by STW 5 administration. The microbial concentration expressed as ng per gram of feces was calculated taking *16S rRNA* gene copy number into consideration as detailed in the Methods section. Data represented as means ± standard deviation of at least two independent experiments with number of animals of at least 14 animals per group

DSS administration led to a 15-fold increase in the *Enterococcus* population of the *Firmicutes* phylum whereas the other three representatives of the phylum, namely, *Clostridium, Lactobacillus,* and *Blautia*, showed a decrease by 23, 73, and 28% respectively (Fig. 3). All the studied representative members of the *Bacteriodetes* phylum showed an increase in the UC model, an effect that was largely prevented by the herbal preparation STW 5 except for *Bacteroides* (Fig. 4). Furthermore, DSS led to a nearly 3-fold increase in the *Actinobacteria* phylum (Fig. 5), but a 500-fold increase in *Bifidobacterium* (Fig. 5). DSS administration led to a 20-fold increase in the relative abundance of *Proteobacteria*, an effect which was significantly reduced to only 3% after STW 5 treatment (Fig. 6). Changes in *16S rRNA* DNA showed an increased level of *Methanobrevibacter* by 2.5-fold (Fig. 7 a) while levels of *Fusobacterium* phylum were not significantly affected by DSS (Fig. 7 b). STW 5 treatment guarded against all the changes induced by DSS.

To gain more insight into the beneficial effects of STW 5 in the DSS model of colitis, it was necessary to show whether the herbal preparation has any effect on the normal microbiota flora or not on its own. The dose of STW 5 (5 ml/kg) have been tested on the microbiota flora in normal rats and was found to be largely insignificant (Supplementary Data).

### Relative abundance of bacterial and archaeal phyla

**Fig. 8:**
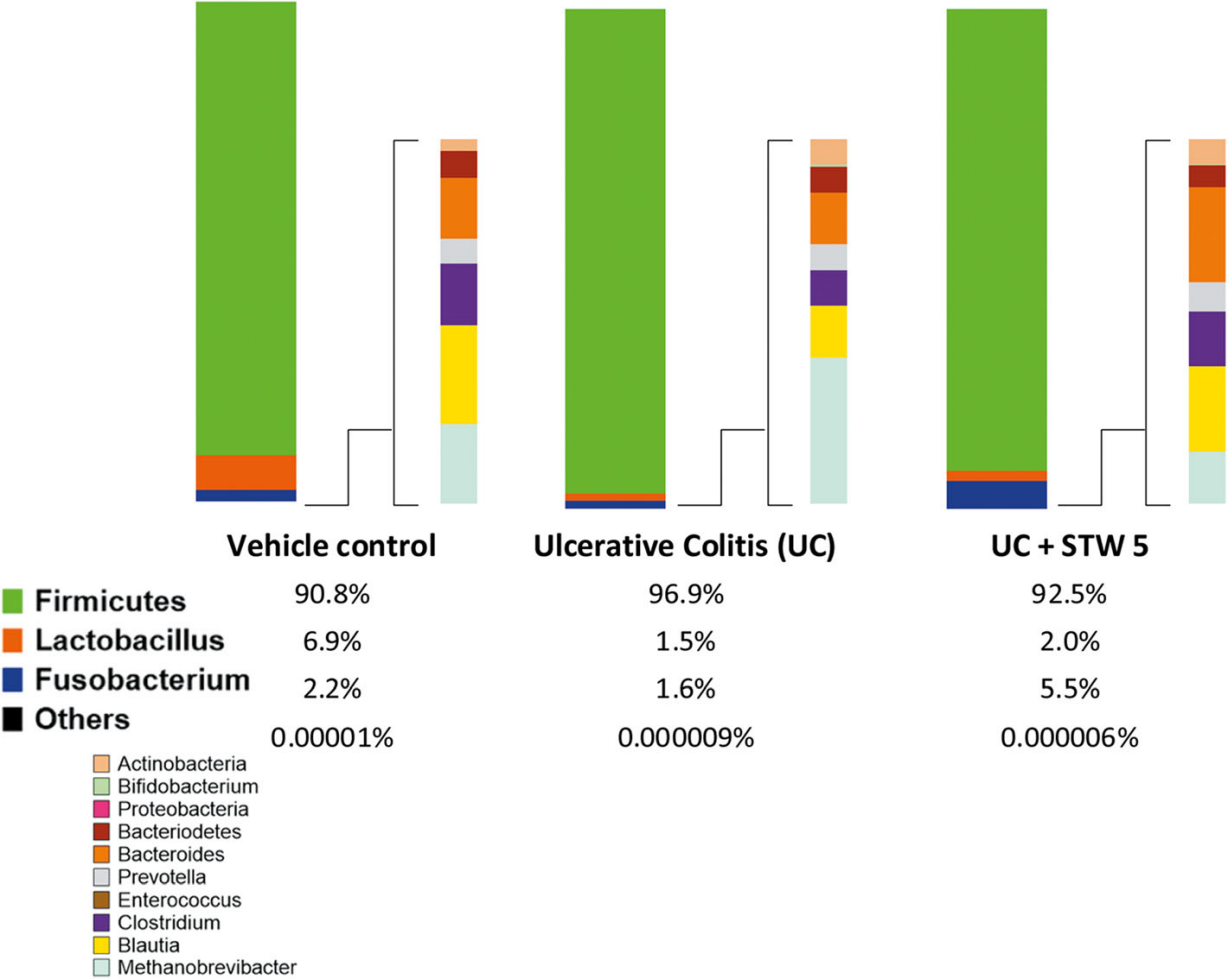
Relative proportions of selected phyla and genera of gut microbiota in vehicle control **A**, UC **B** and UC + STW 5 **C**. The relative proportion of *Firmicutes* as a phylum was increased in UC this was decreased in STW 5 treated group. Within *Firmicutes, Lactobacillus* population showed about 4-fold decrease in UC, STW 5 tended to slightly increase its levels. DSS administration slightly decreased *Fusobacterium* population and increased levels were caused by STW 5 administration. The changes in the remaining phyla and genera of gut microbiota represented less than 0.1% of the total proportional changes observed

To better understand how STW 5 affects the unbalanced microbial community induced by DSS colitis, the relative abundance of measured phyla and genera was analyzed as shown in Fig. 8. DSS-induced dysbiosis was evident by changes in the relative abundance of *Firmicutes, Fusobacterium* and *Methanobrevibacter* phyla and *Lactobacillus, Blautia,* and *Bacteroides* genera. Most of these changes have been resolved by STW 5 treatment, particularly those of *Blautia* and *Methanobrevibacter*.

## Discussion

Gut microbiota is known to maintain a balance between its members to preserve intestinal integrity by preventing pathogen colonization and initiation of inflammation. Derangement of this balance is associated with the development of ulcerative colitis in man [29]. Furthermore, alterations in function and relative abundance of intestinal bacteria belonging to the phyla *Firmicutes, Bacteroidetes*, *Actinobacteria*, and *Proteobacteria* phyla have been implicated in experimental colitis [30]. As well as changes in *Fusobacteria* and members of *Methanobrevibacter* genus [31–33].

In the present study, DSS-induced colitis was associated with variable changes in the different phyla and genera examined. The genera belonging to the Firmicutes showed a five-fold increase in the relative abundance of *Enterococcus* but a significant decrease in that of some commensal microbiota including *Clostridium* cluster IV*, Blautia,* and *Lactobacilli*. These species owe their commensality to the fact that they produce short-chain fatty acids (acetic, butyric, and lactic acids) as fermentation end-products that have an essential role in the metabolic welfare of colonocytes [34–36]. A decrease in *Lactobacillus* has been reported in colonic biopsies from patients with active UC [37, 38].

DSS also induced a dramatic increase in *Prevotella* (phylum *Bacteroidetes*), conforming with earlier reports [39]. Another relevant bacterial phylum in IBD is *Actinobacteria* with its genus *Bifidobacteria.* In the present study, *Actinobacteria* and *Bifidobacterium* levels were dramatically increased by DSS similar to previous studies showing increased proportions of *Bifidobacterium* in UC patients [40]. Furthermore, earlier studies showed an increase in the levels of *Poteobacteria* in DSS-induced colitis [7] as well as in *Fusobacterium varium* in the colonic mucosa of UC patients [31]. Our experimental findings showed similar effects regarding *Proteobacteria* but failed to show significant changes in the *Fusobacterium* population*.*

DSS further raised the relative abundance of *Methanobrevibacter* (phylum *Euryarchaeota*) in accordance with reports that its levels are increased in UC patients and responsible for the bloating and decreased intestinal motility symptoms [41, 42]. STW 5 tended to normalize *Methanobrevibacter* abundance, a fact that might explain the clinically proven efficacy of STW 5 in bloating.

Gut microbiota has been shown to play an important role in intestinal inflammatory conditions, some in initiation and progression of the inflammatory process and some in having an anti-inflammatory effect. For example, *Proteobacteria* has been associated with inflammation in different models of colitis [43, 44] while *Bacteroides vulgatus* has been shown to activate the signaling of NFκB in the gut epithelial cell culture [45]. However, butyrate which is produced by commensal *Clostridia* inhibits NF-kβ activation in gut cells leading to an intestinal anti-inflammatory effect [35]. Furthermore, various strains of *Bifidobacteria* have been shown to exert an anti-inflammatory effect through induction of intestinal IL-10 [40] and treatment with *Bifidobacterium bifidum* was shown to partially protect mice from Th1-driven inflammation in a chemically induced model of colitis [46]. The present findings show indeed that DSS-induced colitis was associated with a marked increase in inflammatory and apoptotic markers such as TNF-α, NFκB, and caspase-3.

STW 5 administration significantly decreased colon inflammation and apoptosis. The anti-inflammatory effect of STW 5 might be attributed in part to decreasing *Proteobacteria* and *Enterococcus* levels and increasing *Clostridia* population, a fact that might help to explain the reduced inflammation and maintenance of the normal bacterial ecosystem. While the administration of STW 5 itself in normal rats had largely insignificant effects on the tested microbiota (Supplementary Data), yet when given to animals with DSS-induced colitis, it significantly decreased the relative abundance of *Bacteroidetes* and *Prevotella* and tended to normalize the abundance of both *Actinobacteria* and *Bifidobacterium* populations. Furthermore, STW 5 increased the abundance of *Bacteroides, Lactobacillus, Clostridium*, *Blautia* as well as *Fusobacterium* and succeeded to normalize *Methanobrevibacter* abundance, a fact that might explain the clinically proven efficacy of STW 5 in bloating.. It is difficult to ascribe the beneficial effect of STW 5 to any one or more of its active constituents. Earlier studies on its gastroprotective effects showed that each individual component contributes to the overall efficacy, but optimal activity was exerted by their combined effects [47, 48]. The preparation as a whole was also shown to be effective in experimental models of functional dyspepsia [49] and colitis [19] as well as clinically in IBS and FD [15, 16]. It would therefore be reasonable to assume that the effects obtained in the present study are the result of the combined activity of the individual components of the standardized preparation.

## Conclusion

DSS-induced colitis was associated with gut microbial dysbiosis, an effect that tends to create a pro-inflammatory milieu, initiating intestinal inflammation. This shift in gut microbial composition included reduced beneficial indigenous microbiota that acts to maintain epithelial health. Treatment with STW 5 showed anti-inflammatory and antiapoptotic effects and favorably affected the intestinal microbiota by decreasing bacteria that contribute to intestinal inflammation as *Proteobacteria* and *Prevotella* and increasing bacteria with anti-inflammatory properties as *Bifidobacteria* and *Lactobacilli*. The results provide an additional novel mechanism of action underlying the beneficial effect of using STW 5 in gastrointestinal disorders.

## Supplementary Information

**Additional file 1: Supplementary data.** Effect of STW 5 (5 mL/Kg) on the microbiome in healthy rats. Microbial population concentrations are expressed as g, mg, μg, ng, or pg per gram of feces (indicated for each microbial population) and calculated taking *16S rRNA* gene copy number into consideration as detailed in methods section. Data represented as means 5 ± standard deviation.

## Data Availability

The raw data generated during the current study are available from the corresponding author upon request.

## Abbreviations

CD: Crohn’s disease
DSS: Dextran sodium sulfate
FD: Functional dyspepsia
FGID: Functional gastrointestinal diseases
GID: Gastrointestinal diseases
IBD: Inflammatory bowel diseases
IBS: Irritable bowel syndrome
NFκB: Nuclear factor kappa B
TNF-α: Tumor necrosis factor-alpha
UC: Ulcerative colitis
