# Supplementary material for: Effect of the standard herbal preparation, STW5, treatment on dysbiosis induced by dextran sodium sulfate in experimental colitis
Source: BMC Complement Med Ther. 2021 Jun 8;21:168. doi: 10.1186/s12906-021-03337-8 (PMC8188707; doi:10.1186/s12906-021-03337-8)
Supplement: Supplementary file 1 — Additional file 1: Supplementary data. Effect of STW 5 (5 mL/Kg) on the microbiome in healthy rats. Microbial population concentrations are expressed as g, mg, μg, ng, or pg per gram of feces (indicated for each microbial population) and calculated taking 16S rRNA gene copy number into consideration as detailed in methods section. Data represented as means 5 ± standard deviation. [file 12906_2021_3337_MOESM1_ESM.pdf]

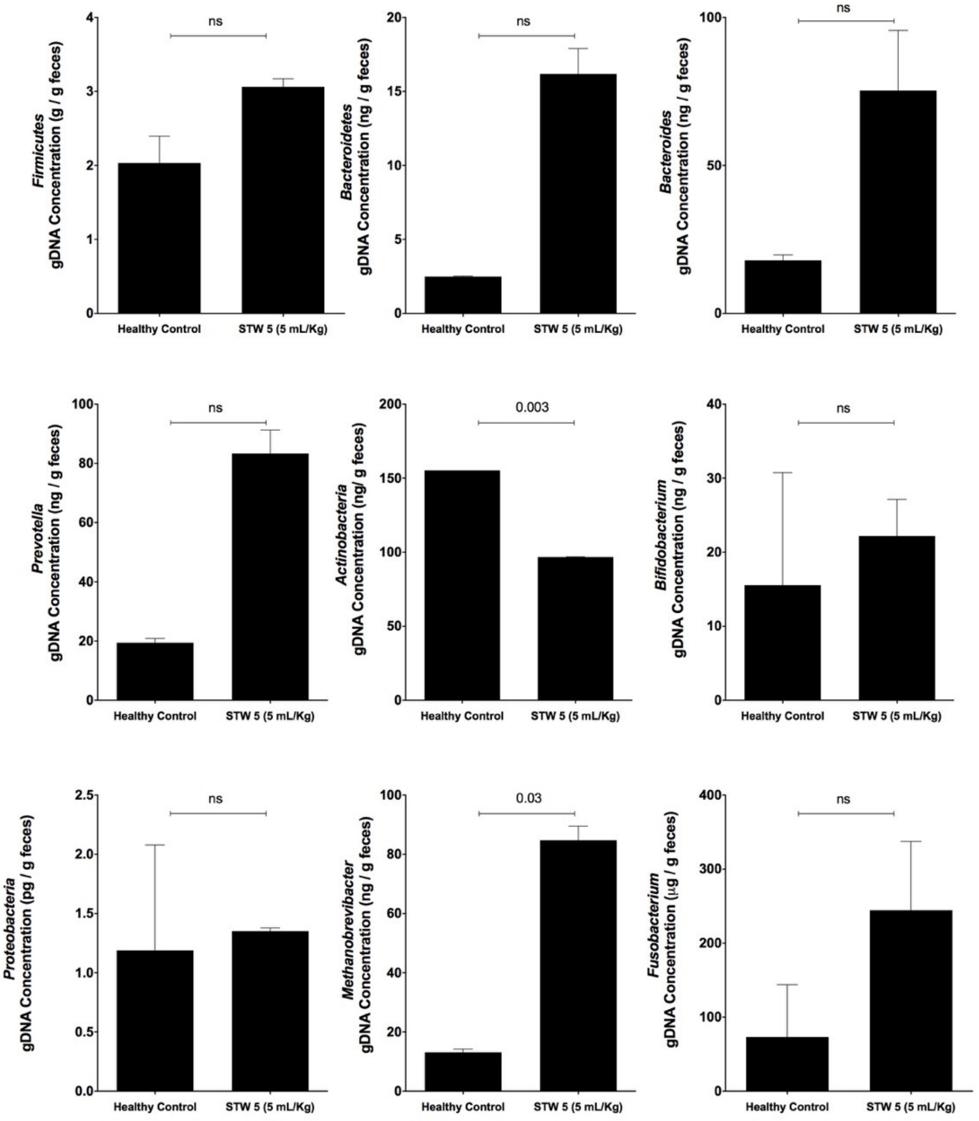

Supplementary data: Effect of STW 5 (5 mL/Kg) on the microbiome in healthy rats. Microbial population concentrations are expressed as g, mg, µg, ng, or pg per gram of feces (indicated for each microbial population) and calculated taking 16S rRNA gene copy number into consideration as detailed in methods section. Data represented as means ± standard deviation.
